# Bringing the voice of social housing tenants into shaping the health and care research agenda

**DOI:** 10.1186/s40900-024-00613-y

**Published:** 2024-08-08

**Authors:** Olivia R. Phillips, Denise Mardell, Kolin Stephenson, Sabrina Hussain, Dawn Burton, Barbara Bernard, Sue Stevenson, Joanne R. Morling

**Affiliations:** 1 Nottingham Centre for Public Health and Epidemiology, Lifespan and Population Health, School of Medicine, University of Nottingham, Nottingham, UK; 2 NIHR Nottingham Biomedical Research Centre (BRC), Nottingham University Hospitals NHS Trust, University of Nottingham, Nottingham, NG7 2UH UK; 3 The Together Network, Nottingham, UK

**Keywords:** Social housing, Public and patient involvement, PPI, Public health, Health inequalities, Research partnership

## Abstract

**Background:**

A larger percentage of social housing tenants have poorer physical and mental health outcomes compared to private renters and homeowners. They are also at a greater risk of respiratory conditions, cardiovascular disease, communicable disease transmission and mortality. One approach that aims to reduce health inequalities is to create research partnerships with underserved local communities. Our primary aim was to develop a research partnership with social housing tenants in Nottingham and our secondary aim was to explore the health priorities of these social housing tenants to inform future research applications. We also hope to provide a descriptive process of PPI within a social housing context for other researchers to learn from.

**Methods:**

We used Public and Patient Involvement (PPI) as the foundation of this work, as we believed that people with lived experience of social housing, also end-users of the research, were best placed to inform us of the areas with the greatest research need. Through online and in-person focus groups, we discussed with tenants, collectively named a Social Advisory Group (SAG), their health concerns and priorities. Together they raised 26 health issues, which were combined with 22 funding opportunity themes being offered by the NIHR (National Institute for Health and Care Research). This was with the purpose of investigating whether there was alignment between the health needs of Nottingham’s social housing tenants and the NIHR’s research priorities. A prioritisation technique (Diamond Nine) was used to sort in total, 48 areas of health and wellbeing, into three top priorities. Tenants were provided the opportunity to be involved in public health research in other ways too, such as reviewing this paper and also an NIHR Programme Development Grant application to expand and continue this work. One was also offered the opportunity to be a public co-applicant.

**Results:**

The group prioritised improvements in the quality of social housing, mental health and healthcare services. There was only some alignment between these and the NIHR funding themes. Other factors, such as age and race, also determined individual health priorities. . The diversity and reach of the current project were limited, however this is something we hope to improve in the future with more funding. We learned that tenants have varying degrees of mobility and technological abilities, requiring both online and in-person meetings.

**Supplementary Information:**

The online version contains supplementary material available at 10.1186/s40900-024-00613-y.

## Abstract (plain language summary)

Social housing is offered to people who cannot afford to buy or rent in the open market, and a larger percentage of social housing tenants have poorer physical and mental health outcomes compared to the general population. One approach that aims to reduce health inequalities is to create sustainable research partnerships with underserved local communities. Our primary aim was to involve social housing tenants in public health research, as they are best placed to tell us the type of research they would benefit from. The secondary aim was to explore the health priorities of social housing tenants to inform future research applications. We also hope to describe the process of PPI within a social housing context for other researchers to learn from.

To achieve these aims, we established a research partnership with a group of social housing tenants in Nottingham and spoke to them about the areas of their health they wanted to improve (i.e., their priorities). The topics that were discussed the most were the need for improved mental health, quality of social housing and healthcare services, however this varied between individuals according to race and age. We learned several things throughout this process. Firstly, the combination of mobility and technological abilities amongst tenants meant that meetings must be held both in-person and online. This ensured they remained accessible and convenient. Secondly, we learnt that in-person meetings should be held in a neutral space to encourage different members of the group to attend. Finally, in general, people were very enthusiastic about this partnership and were committed to seeing improvements in public health. We therefore provided more opportunities for the group to be involved in research. For example, they were offered the opportunity to write and edit a lay summary for a future research application, which was based on the priorities identified in this paper. One member of the group was nominated to be the public co-applicant , which would allow us to increase the reach of this housing work across the East Midlands. It would also allow us to increase the diversity of the group, as currently it is made up of mostly retired females of British origin. Involving the public in health research has been central to this process and continues to be important in the production of accessible and relevant research.

**Supplementary Information:**

The online version contains supplementary material available at 10.1186/s40900-024-00613-y.

## Background

### Social housing

Social housing is offered to people who cannot afford to rent or buy a home in the open market. This type of housing can be rented from councils or housing associations (HA) at a decreased price for people with low incomes, or it can be part-sold or part-rented as shared ownership [1]. In the UK, housing is allocated based on a needs assessment, where, generally, people are prioritised for housing if they are homeless, have a medical condition exacerbated by current housing or live in cramped conditions (although there are variations by local council/nation) [2]. In 2016–2018, 3.9 million households in England (17%) lived in social housing [3] and there continues to be high demand which is not being met. The UK is in a housing crisis, with 2022 data showing that 1.21 million households were on a local authority waiting list for social housing in England alone [2].

However, sometimes social housing is the preferred choice. For example, it is common that younger tenants move into council-owned properties to gain the Right to Buy, thus giving them a chance to get onto the property ladder. This provides tenants the legal right to purchase their house at a large discount. Similarly, a generational attitude exists, whereby living in social housing is considered normal due to an individuals’ family having a history of renting council-owned properties.

### Housing and health

Housing is an established social determinant of health [4]. Approximately one third of people renting from a HA or local authority (LA) experience mental health issues, compared to 25% for private renters and 20% for homeowners [5]. This poorer mental health (upset, frustration, depression, anxiety) has been attributed to the conditions of social housing [6] through, damp, leaks, overcrowding, crime, antisocial behaviour and delays in seeing to repairs, which was sometimes years [7–10]. Further, experiencing antisocial behaviour caused decreased feelings of belonging and safety [8].

Housing insecurity also contributes to poorer mental health. This is associated with tenant’s relatively low incomes. The average weekly income for general needs social housing tenants in England in 2021/22, including pensions and benefits, was £254, compared to >£600 for the UK general population [11]. Introductory tenancies can also contribute to housing insecurity, whereby new tenants undergo a ‘trial’ period for 12 months before becoming a flexible or secure tenant. During this time, renters can be removed if they break their terms and conditions [12].

Poor quality social housing can negatively affect physical health. Damp, cold, noise and mould have been associated with increased respiratory conditions, cardiovascular disease, communicable disease transmission and mortality [13, 14]. Overcrowding can cause sleep issues and conflict amongst families, as well as a lack of space for children to play and study, leading to poorer performance at school due to limited study space [10]. Many of these factors are beyond the control of tenants, indicating that this is a systemic inequality disproportionately affecting low-income individuals and families, and thus, those living in social housing.

### Previous involvement of social housing tenants in research

Research of social housing tenants, where tenants are involved in the research process as members of a PPI group, is limited. Individuals who engage in PPI typically report age, sex, ethnicity and other basic demographic information, therefore social housing tenants may be included but researchers will not know. This demographic can also be harder to access due to greater work responsibilities, significant caring responsibilities or disability, therefore a lot of resources are needed to accommodate this group. For this research, we defined PPI as, “Research that is done “‘with’ or ‘by’ the public, rather than ‘to,’ ‘about’ or ‘for’ them” [15]. We identified two UK studies potentially involving social housing tenants in their research development; however, their exact involvement was unclear [16, 17].

The current research aimed to rectify this at a local level, with the research agenda being set entirely by social housing tenants. This will go on to influence future grant applications, which will address areas of research need identified by its end users. The approach we have taken with our tenant group and this PPI process as a whole, is based upon peer partnership models. These are grounded in the premise that both community members and researchers learn from each other to promote “hybrid knowledge” between the community and academia [18]. These models emphasize the importance of researchers collaborating with “insiders”, or lay community members, united by one or more characteristics e.g., language, values/norms, experiences, context etc. Working in light of these likenesses is beneficial, as it encourages trust between peers, who also appear more approachable due to their shared insider status [19]. In turn, this positively impacts research as these peers can connect academic researchers to the community of interest through shared, local knowledge and similar lived experiences. Instead of being people to give advice, peer partnership models consider lay community members, or social housing tenants in this case, as individuals to work alongside. This is beneficial over employment models (where peers are hired for specific tasks) or advisory models (where peers provide guidance and support to researchers), as partner models increase the relevance of the research in question and the sense of ownership experienced by community members [19].

### Aims

Our overall aim was to build capacity and provide sustainable opportunities amongst underserved populations to be involved in research. This was broken down into two more measurable aims. The primary aim was to establish a public involvement partnership with social housing tenants. The secondary aim was to explore the health priorities of social housing tenants to inform future research applications. We also hope to provide a descriptive process of PPI within a social housing context for other researchers to learn from.

## Methods

### Providing opportunities and building capacity amongst social housing tenants to be involved in research

We involved social housing tenants in public health research through a research partnership, which was enabled through our pre-existing relationships with two housing associations: Nottingham City Homes, now Nottingham City Council Housing Services (NCCHS) and Nottingham City Housing Association (NCHA). NCHA manages 10,000 homes in Nottinghamshire, Derbyshire, Lincolnshire, Leicestershire, Northamptonshire and Rutland, providing homes to over 20,000 people. NCCHS is larger, with 26,500 homes in Nottingham and over 63,000 people living in them.

**Fig. 1:**
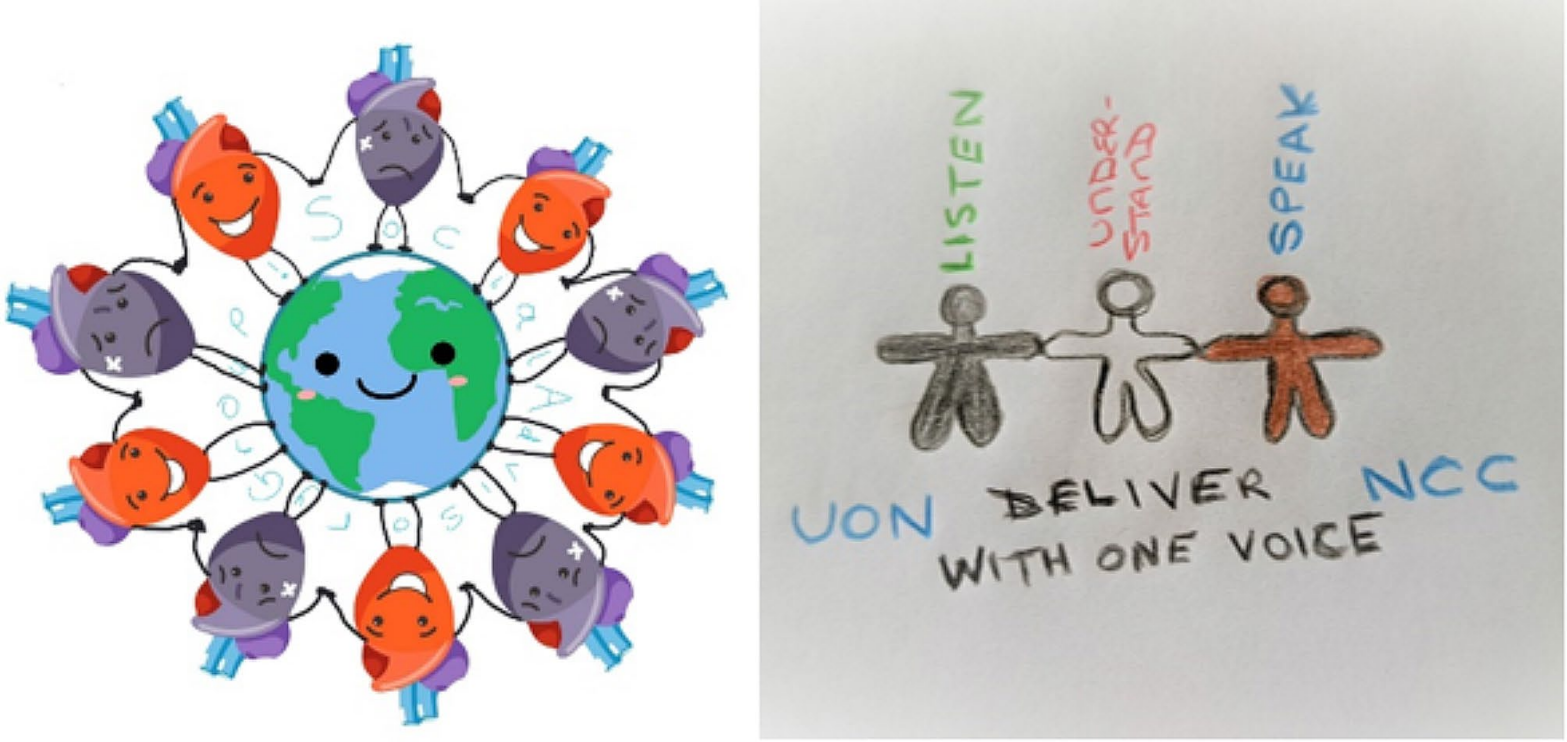
Logos created by two members of the SAG

Through contacts at NCCHS and NCHA, tenants were made aware of this public involvement opportunity via email, social media and tenant newsletter. This opportunity was also promoted at social housing events visited by the researcher. This included at the end of a diabetes awareness session and at a lunch club in Bulwell - a weekly social club where housing tenants come together for games and lunch, which they can enjoy at a discounted price (approximately £2).Tenants were informed that we were conducting research into the health priorities of Nottingham’s social housing tenants and that we would like their involvement as co-researchers using their lived experience. The invitation included information on the reimbursement of travel costs, provision of training to enable participation (e.g. how to use Microsoft Teams) and the choice of virtual/physical meetings. We offered to cover childcare costs and ensured tenants that venues would be accessible. Translation services were also offered but no one expressed a need for this. Tenants received £20 per meeting as a recognition of their contribution, either through bank transfer or Amazon/Love2Shop voucher. This information was also included in the invitation. Recruitment commenced in January 2023 and was ongoing until April 2023. Thirty-two tenants expressed interest in being part of this research opportunity. Those who signed up were asked to indicate the most convenient times for them to attend meetings. At their request, the group was named a Social Advisory Group (SAG) to reflect their position as social housing tenants. To strengthen the identity of the group, we asked them to create a logo to bring together the concepts of social housing, health and community (see Fig. 1). To ensure ongoing engagement with the group, tenants were sent a newsletter once every two months with updates about the project’s progress and a summary of the meetings that had taken place. Five members of the group also contributed to the reviewing and editing of this paper and have been named as co-authors.

We have since applied for further funding to expand this community partnership across the East Midlands. This NIHR funded grant provided more opportunities for the SAG to be involved in research (see ‘Discussion’ for further details), titled ‘Improving access to public health research in underrepresented populations: a research partnership with social housing organisation in the East Midlands’. This is a partnership of social housing tenants, their providers, local decision makers and academics, and has been informed by the work in the current paper. To establish the network, we will identify economically disadvantaged places where research is rarely carried out and create partnerships with local organisations there. This will allow us to assess how feasible it would be to deliver research in that area and to identify members of the public and staff who are willing to help do this. Once this network is established, we will have several workshops with all partners that will decide together: the network’s aims and expectations, research priorities and questions and any training needs. When we discussed this grant with members of the SAG, they suggested the network be titled, ‘The Together Network’, which we took onboard. They also helped write the plain English summary for the proposal and gave feedback on its content. One member of the SAG is also supporting as public co-applicant. This member will be the voice of the public throughout the research. They can offer a different perspective from other members of the team, who will mainly be from an academic or public health background. They will attend team meetings and provide input on study design and delivery using their lived experience and will also help co-ordinate and deliver public involvement activities.

Whilst establishing the community partnership, the primary researcher kept a “learning log”, detailing anecdotal evidence and reflections of the strengths and challenges of the process. In addition, the group was sent a feedback survey asking about their experiences of being part of the SAG. This allowed us to reflect on the process using Liabo’s [20] established framework for evaluation of public involvement.

### Identifying priorities to shape future bids

To establish which aspects of health and well-being were considered a priority for tenants, first there was a need to create a pool of health and wellbeing issues from which these would be chosen. Six meetings were held (three online and three in-person in communities), between March and August 2023. Attendees included social housing tenants from both NCHA and NCCHS and a researcher from the University of Nottingham. To build rapport within the in-person groups we used ‘pen portraits’, whereby attendees created short descriptions of themselves, such as their hobbies, life motto and skills they could bring to the group (see Additional File 1). Some sessions utilised existing group activity meetings, such as the lunch club in Bulwell. This meant that discussions were broken down into informal conversations with one or two people at a time when appropriate. The three online meetings were conducted over Microsoft Teams. During these meetings field notes were taken by the researcher, and they were also audio recorded. The meetings were held in English; therefore, some level of proficiency was required.

Discussion was prompted by questions such as, “What does health and wellbeing mean to you?”, “Is health and wellbeing a priority?” and “What areas of your health and wellbeing would you like to improve?”. Group members were informed that their answers could relate to any aspect of health, such as physical or mental health, their environment, social wellbeing etc. Tenants discussed, related, and shared stories and experiences with prompts and probing by the researcher. In the online meetings, a Padlet was available for tenants to use to write their health issues should they not want to voice them; however, conversation was preferred. The result of these meetings was a list of 26 health issues brought to light by 32 social housing tenants. This list was then sent back to those who attended the meetings to verify that the researcher’s interpretation was correct.

**Fig. 2:**
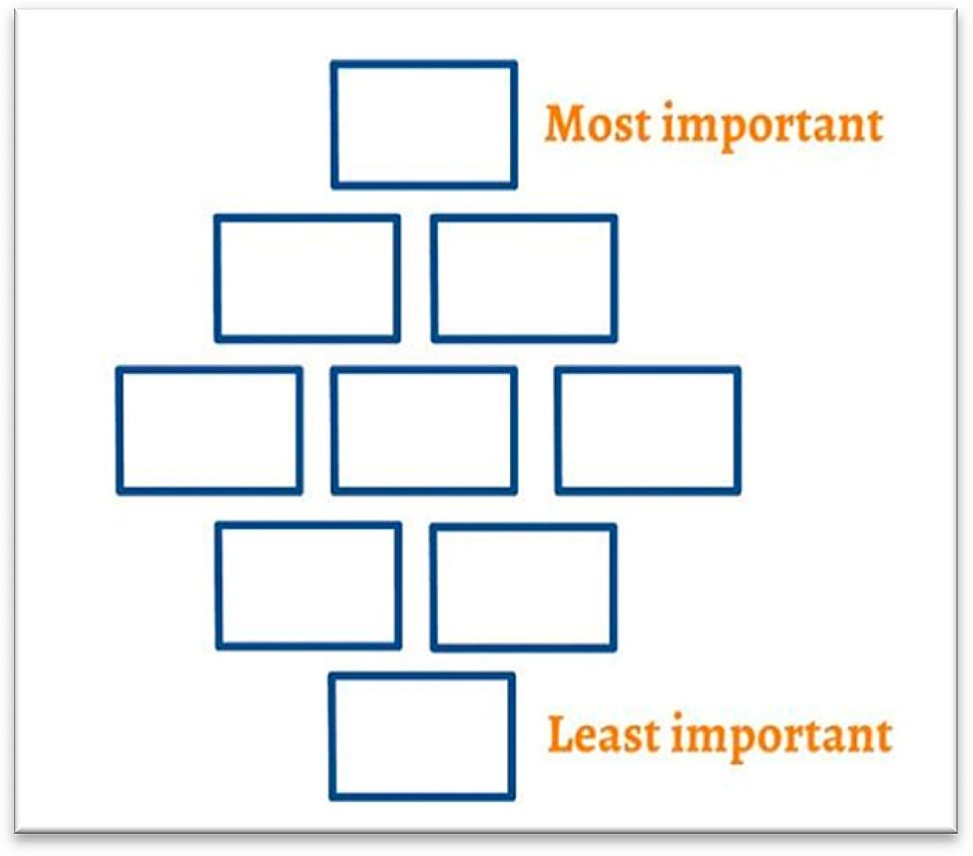
The Diamond Nine prioritisation template

The 26 health issues were combined with 22 NIHR Public Health funding opportunities (as of July 2023) [21] (see Additional File 2). This allowed us to investigate whether the topics that were receiving research funding aligned with the health priorities of social housing tenants. This meant there was a total of 48 health issues. One in-person (*n* = 7, local library setting) and one online (*n* = 5) meeting was held to refine this list. For the in-person meeting, the health issues were printed onto individual cards and sorted using a prioritisation technique (Diamond Nine) (see Fig. 2) [22] along with the opportunity to add further topics if not included. The group was informed that some of the cards were NIHR funding themes but were not told which ones. Tenants worked together in groups to identify their nine most important issues into a diamond. The card at the top was considered the most important and the card at the bottom the least. The groups then expanded on their top three priorities by considering, “What would it mean to you to have this [their top three issues] improved?” and “Why is this a problem for you?”. All but one who attended the prioritisation discussions had attended previous meetings to identify health and wellbeing issues. The same prioritisation activity was emailed or posted to the remaining members of the group who did not attend the in-person or online meetings. Three members responded. Throughout this process, meetings were never held at the university as we did not believe this was conducive to equal power dynamics.

## Results

### The SAG

In total, 32 social housing tenants signed up to be a part of the group, however only 15 people contributed to the prioritisation discussions. Further details can be found in Table 1.

**Table 1:** A table displaying the number of tenants who signed up to the SAG and participated in the Diamond Nine activity

	Signed up to be a member of the SAG	Completed the Diamond Nine
*N* (%)	32 (100)	15 (47)
Gender		
Female	27 (84)	12 (80)
Male	5 (16)	3 (20)
Housing Association		
NCCHS	21 (66)	11 (73)
NCHA	5 (16)	2 (13)
TunTum	1 (3)	1 (7)
Other	5 (16)	1 (7)
Age		
25–34	1 (3)	1 (7)
35–44	6 (19)	3 (20)
45–54	2 (6)	0 (0)
55–59	5 (16)	3 (20)
60–69	10 (31)	4 (27)
70–74	2 (6)	2 (13)
75–79	3 (9)	2 (13)
80–84	2 (6)	0 (0)
Prefer not to say	1 (3)	0 (0)
Sexual orientation		
Bisexual	1 (3)	0 (0)
Gay man	1 (3)	1 (7)
Heterosexual	26 (81)	11 (73)
No response	0 (0)	1 (7)
Prefer not to say	4 (13)	2 (13)
Ethnicity		
African	1 (3)	0 (0)
African Caribbean	1 (3)	0 (0)
Asian/Asian British Indian	1 (3)	0 (0)
Black British	1 (3)	0 (0)
Black Caribbean	1 (3)	0 (0)
Caribbean	1 (3)	1 (7)
Pakistani	2 (6)	2 (13)
Prefer not to say	2 (6)	0 (0)
White and black Caribbean	3 (9)	1 (7)
White British	18 (56)	11 (73)
White Irish	1 (3)	0 (0)
Do you consider yourself disabled?		
Yes	16 (50)	6 (40)
No	12 (38)	6 (40)
Prefer not to say	4 (12)	3 (3)

### Health priority findings

**Fig. 3:**
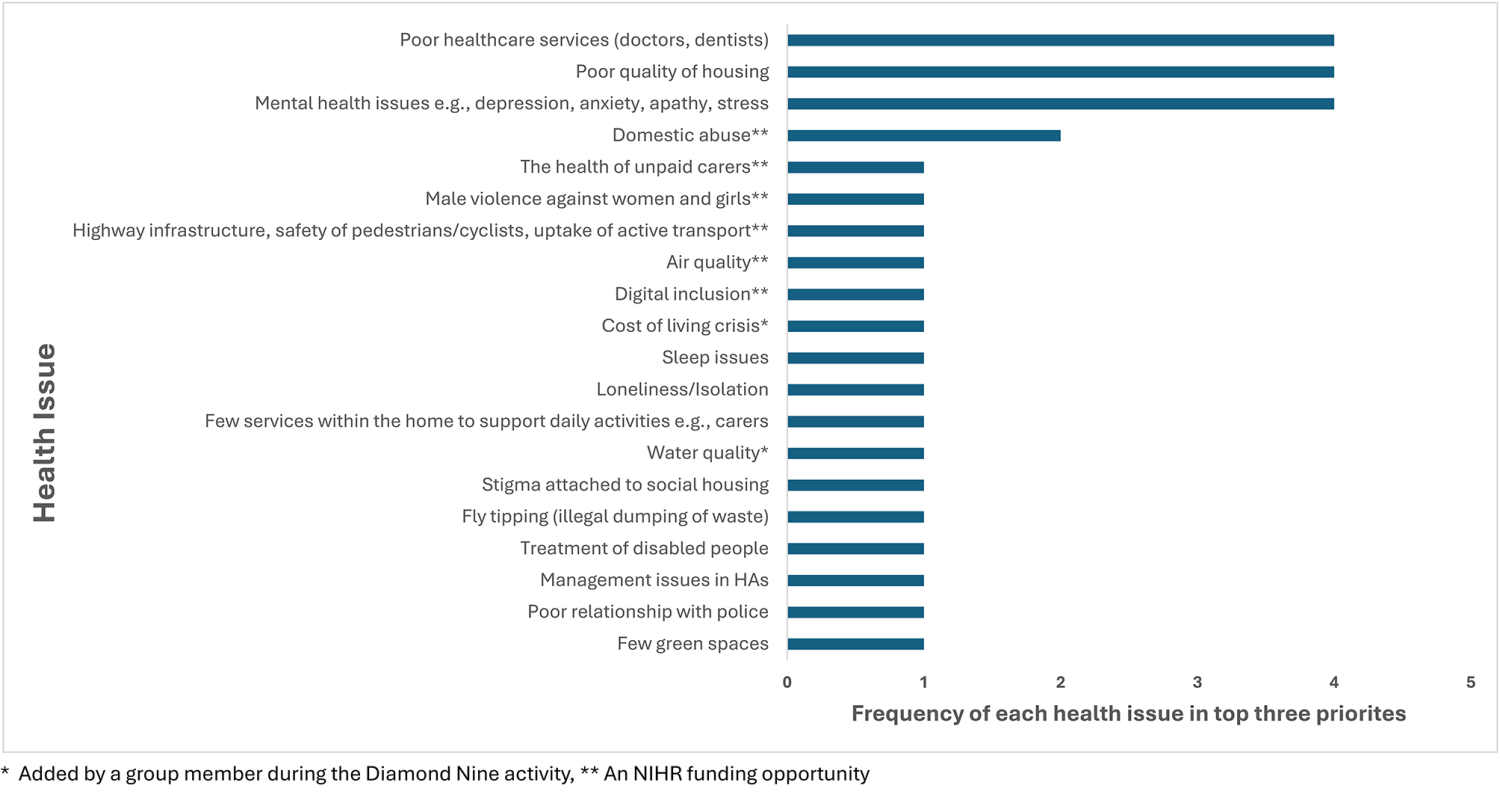
A bar chart displaying the frequency of which the health issues were placed in the top three priority spaces

Overall, there were 10 completed diamonds from 15 individuals. Five from online members of the group, two from in-person participants and three postal responses. Twenty different issues were placed within the top three priorities spaces in the prioritisation activity (Fig. 3) with the majority being those initially raised by tenants. The health issues rated as most important, which were each allocated a top three place four times, were poor quality of housing, poor healthcare services, and mental health issues.

Firstly, poor quality of housing was cited to negatively impact tenants’ health and wellbeing. This included the negative impacts of damp, mould and their mycotoxins as well as long waits for repairs. Secondly, tenants believed that the NHS is lacking the resources to provide high quality care, citing it to be an overwhelmed system with a lack of consistency, communication, empathy, compassion, continuity of care, appropriate services and time with practitioners. Waiting times were an issue, both within the context of A&E as well as for appointments for mental health services, physiotherapy and dentistry. The lack of resources within the healthcare system led tenants to feel unimportant and unheard. They called for increased funding and improvements in staff people skills. Tenants expressed that they would feel acknowledged and more confident to access help if these issues were resolved. Thirdly, the poor mental health status of the population was again, related to strained resources. People experienced busy phone lines, a lack of councillors/therapists and support, as well as long waits for appointments, so much so that people had “given up” and deemed the process “pointless”. Specifically, the lack of education about mental health and coping strategies was cited as a problem. People specifically wanted to be treated as an individual, rather than a statistic, and voiced that losing their benefits due to mental health issues is a worry. Regarding alignment to the NIHR funding themes, none were consistently placed in the top three priority spaces.

### Group evaluation

Eight tenants completed online/postal feedback surveys. This survey was based upon an evaluation form developed by Imperial College London [23], however was adapted to include items specific to the current research. All felt like they were well-informed about the project and that the different meeting options were helpful. People liked meeting others, hearing their views and concerns, talking openly and feeling like they were being listened to. However, some believed that other members were there for the money and did not contribute to discussion. Whilst the flexibility around meeting options was considered a positive by some, for others not knowing who would be at future meetings and that the attendees often changed was a barrier to attendance. Tenants felt as though communication (emails/newsletter) from the researcher was clear, useful and frequent enough to be informative but not so frequent that it was irritating. However, one felt as though there was sometimes a long pause between emails. When asked what they had gained from this process, members experienced enhanced knowledge about the research process, improved written and spoken language skills and research and communication skills. One was reminded how important it is to listen to people who are unheard. Another reported learning about the “bigger picture” and that they should learn to be more tolerant. The chance to listen to others from all walks of life, as well as knowing that they are not alone in their feelings, were also take-aways from this process. All respondents believed their input had made a difference to the process and overall, it had a positive impact on public contributors.

This feedback (as well as the researcher’s reflective learning log) allowed us to use a more established framework to evaluate the PPI. A literature search by Liabo et al. (2020) [20] identified five values and seven practicalities to quantify ‘good’ public involvement. Values are an ideal of importance to public involvement and a practicality is something that enables a value. Overall, this work fulfilled most values and practicalities identified by Liabo et al. (2020) [20], however there are still improvements to be made (see Table 2 for more a detailed description on how the values and practicalities were met/not met.)

**Table 2:** PPI values and practicalities identified by Liabo (2020) [20]

Value principles	Definition	How it was met / not met in the current study
Inclusivity	Involvement of a diverse range of people, and equal opportunities for people to become involved irrespective of their social backgrounds and abilities	Although the project was open to everyone from all backgrounds, many members of the SAG were of a similar demographic, therefore we cannot say with confidence that ‘inclusivity’ was met.
Partnership	Researchers and involved public members showing respect for each other’s contributions and roles and working together in teams.	There was genuine collaboration between the public, who drove the research direction, and the researcher, who organised, facilitated and guided meetings, which were harmonious and productive.
Purposeful involvement	Clarity on why members of the public are involved in research and this is communicated to everyone involved. There is a commitment to involvement.	The SAG was informed of their role, the time commitment and the potential outcomes of the project prior to their involvement. They were told social housing tenants are underrepresented in research and that their involvement was key in creating tailored research. All members who completed the feedback survey said they felt well-informed prior to attending meetings.
Transparency	Open and honest communication between researchers and public advisors, and clarity on why things are done in certain ways.	The SAG was kept up to date on project progress via email and newsletter. They were made aware that research activities would decrease due to finite funding and the subsequent application for more. Members were also told that hybrid meeting styles would be implemented to cater for different needs.
Value different kinds of knowledge	Recognises that public advisers have complementary expertise to researchers’ technical knowledge.	The public acknowledged the researcher’s professional expertise whilst the researchers acknowledged their expertise by experience. There was an implied awareness that both needed the other to create meaningful outcomes.
**Practicalities**		
Support to public advisors	A budget for reimbursing travel and time, and dedicated staff who attends to individual needs before, during and after meetings (for example; vision aids, disability access), and who advocate for involvement within the research institution.	The group was given financial reimbursement for their time and travel costs were covered. We also offered to cover childcare costs and ensured accessible venues for meetings.
Capacity building	Co-learning between public advisors and researchers, and training for both groups.	Although training (e.g., digital upskilling) was offered to the group, it was not taken up, which makes it difficult to assess whether the ‘capacity-building’ practicality was met. The research facilitator attended a course on PPI within research during this process.
Proportional involvement	Involvement tailored to the needs of the research and public advisers, and pragmatic decisions are made to balance contradicting demands and limited resources.	Members were reassured that we were not expecting this research to be their priority and that we aware they had other responsibilities (e.g., childcare, employment). Currently, this is a small research study, reflected in the minimal time commitment we asked of the group.
Communication	Closely linked to the principle of support, and needs to be responsive and proactive. Public advisers need to be updated on how the research progresses, and the communication mode should suit the needs of public advisers.	The group was sent newsletters every two months with project updates and were also sent emails regarding meetings. Where members were not digitally active, communication was via post.
Involvement throughout the research	There should be opportunities to be involved as a patient, carer or public adviser in any stage of the research. Involvement throughout the research is practically enabled by having infrastructure, leadership and a governance framework for public involvement	The SAG created the list of health issues and decided the research priority. They also contributed to a grant application, and one became its public co-applicant. Group members were invited to help write the newsletters. Five members also reviewed and edited this paper.
Evaluation	Identifying good practice, through communication, research and learning from each other	An evaluation survey was sent to the group after the last meeting was held. We received feedback which will help shape future research activities.

## Discussion

This PPI work has highlighted the varied health priorities of a selection of Nottingham’s social housing tenants, with a greater consensus being reached on three issues, namely quality of housing, the healthcare system and mental health issues. The fact that no NIHR funding themes were consistently placed within the top three priority spaces might suggest that existing funding did not reflect the needs and priorities of the community., and that there is greater research need in other areas. However, it could be suggested that there is some overlap, e.g., ‘the mental health and well-being of young women’ and ‘suicide prevention’ themes offered by the NIHR is related to the broader ‘mental health’ theme raised by tenants. Similarly, the ‘eviction and homelessness’ and ‘uptake of welfare benefits and vouchers’ offered by the NIHR could relate to the broader ‘quality of housing theme’, which suggests that funding is being allocated to areas of need.

It is likely that the prioritisation of ‘poor healthcare services’ reflects group’s position as general healthcare service users, rather than social housing tenants. The NHS, which provides care for 1.3 million people daily [24], is underfunded and lacks appropriate resources to provide the highest quality care. Interestingly, although there was a degree of consensus concerning the top three priorities, there were some notable individual differences. One woman in her seventies placed ‘loneliness and isolation’ as her top priority and a group of four from the in-person meeting, where three of the members were aged between 70–79, placed ‘digital inclusion’ at the top of theirs. A younger female tenant thought that male violence against women and girls was a priority, an elderly lady who often uses a wheelchair thought that the treatment of disabled people was a priority, and a black woman thought that the inappropriate use of oximeters tested on white skin but used on black skin was a priority. This demonstrates that although there was some agreement, people have individual needs defined by characteristics other than their identity as a social housing tenant, such as age, gender, ability and race.

It is possible, however, that the top three priorities listed by tenants are interrelated. For example, poor quality housing creates or exacerbates mental (and physical) health issues, which are challenging to resolve due to the strained healthcare services. It could be suggested that the commitment from the government to improve social housing in general may be the key to reducing health and wellbeing issues in this population, and in the long run, reduce pressure on health services.

### Relation to previous literature

The opinions voiced by tenants in the current paper are similar to those in the literature. Qualitative research conducted in both London and Northern England supports the link between mental health issues and poor quality of housing, namely the presence of mould and damp, the delays in repairs and further deterioration in health [7–9]. Similarly, research conducted on a large cohort of lower-income Australians found an inverse association between time spent in social housing and mental health [25]. That similar findings have been found in different geographical locations may suggest a degree of generalisation for this health priority and highlights the need for improved social housing, both in quality and quantity, which is not restricted to the UK. This is exemplified by the Grenfell Tower fires and Awaab Ishak, a two-year-old boy who died due to mould exposure in the one-bedroom flat he shared with his parents. In response to these events, the English government committed to improving the social housing sector [26].

Contrasting research by the University of Birmingham has found that social housing can positively benefit health and wellbeing [27, 28]. This three-year survey study in East Devon found that suitably allocated and managed social housing can ease anxiety in comparison to privately rented accommodation. This is especially true for people with mental health issues. Also associated with social housing was life satisfaction, a sense of control and feelings of safety and privacy within the home [27]. This suggests some degree of individual and regional differences exist and that social housing has potential to succeed, so long as it is managed appropriately. Further, research with over 200 social housing tenants from across England revealed that stigmatisation from living in social housing was common [29]. However, this was only considered a top three priority once in the current PPI work. This might reflect individual differences; however, it may also imply that the current group was not as representative as it could have been of the wider social housing community.

### Practical and research implications

The current research has demonstrated the value of meaningful co-creation with members of the public. They have provided relevant research ideas and unique insights into areas of social housing that researchers do not possess, which has already helped shape a grant application to expand this community partnership across the East Midlands. This NIHR funded grant provides more opportunities for the SAG to be involved in research. It is titled ‘Improving access to public health research in underrepresented populations: a research partnership with social housing organisation in the East Midlands’. This is a partnership of social housing tenants, their providers, local decision makers and academics. It has a focus on mental health, quality of housing and healthcare as per the findings of the current paper. For example, the application includes the implementation of mental health screening among social housing tenants and the improvement of housing quality to test the impact on mental (and physical) health. To establish the network, we will identify economically disadvantaged places where research is rarely carried out and create partnerships with local organisations there. This will allow us to assess how feasible it would be to deliver research in that area and to identify members of the public and staff who are willing to help do this. Once this network is established, we will have several workshops with all partners that will decide together: the network’s aims and expectations, research priorities and questions and any training needs. When we discussed this grant with members of the SAG, they suggested the network be titled, ‘The Together Network’, which we took onboard. They also helped write the plain English summary for the proposal and gave feedback on its content. One member of the SAG is also supporting as public co-applicant. This member will be the voice of the public throughout the research. They can offer a different perspective from other members of the team, who will be from an academic or public health background. If the application is successful, they will attend team meetings and provide input on study design and delivery using their lived experience. They will also help co-ordinate and deliver public involvement activities. We aim for 'The Together Network to involve a more diverse range of people, both in demographics and region, however this has not yet commenced due to funding constraints, but the current research provides a strong starting point. Further, although there were three topics in the priorities discussions that gained more attention than others, in general there was great heterogeneity in responses (see Fig. 3). These are all important areas of improvement, which future research, such as ‘The Together Network’, could explore.

The effective use of PPI also ensured that people were engaged with the process and have a sense of control over research that might impact them. We encourage other researchers to adopt a similar approach to enhance their output and ensure relevance to the target population. This group of tenants, however, had little to no research or PPI experience, therefore they initially relied on the researchers to guide the process, which decreased as time went on and their confidence increased. In the future, we will encourage confidence in SAG members from the start. To accommodate this, we will continue to offer training, whether that be to familiarise members with the research process or to improve digital capabilities.

We would still agree with the definition of PPI provided at the beginning of this paper, that “Research is done “‘with’ or ‘by’ the public, rather than ‘to,’ ‘about’ or ‘for’ them” [15]. We would, however, suggest that the term ‘co-researcher’ accompanies this, as we believe it solidifies the public’s role alongside researchers in academia and provides them equal status.

This research compliments and supports the peer partnership model. Due to the level of involvement of the SAG, engagement was sustained by a core group and we were able to gain an understanding of community needs. Hybrid knowledge was created and mutual learning took place, whereby researchers discovered more about the health and wellbeing of social housing tenants, and social housing tenants learned of the research environment. This is supported by results of the evaluation survey, where respondents said they had enhanced knowledge of the research process, improved written and spoken language skills and research and communication skills. Others learned about the “bigger picture” and learned to improve their tolerance of people. This supports the use of partnership models over employment or advisory, where the same outcomes may not have been achieved due to a more limited sense of involvement and ownership.

### Lessons learned and strengths

There were initial challenges explaining the group’s role and the aims of the partnership as most tenants had little to no research background. Following poor turnout at the first in person meeting (where we asked the tenants to travel to a community venue) we changed tactic and instead we travelled to meet them (e.g. the Bulwell lunch club). This was more effective and convenient for tenants, and most of the group signed up during these visits. Further, over time it was clear that the larger group of 32 individuals was composed of subgroups defined by their meeting preferences, mobility and technological abilities. This meant the group was divided into, generally, an online group, an in-person group and a group from the lunchtime club in Bulwell. This may not be a limitation, however, as it reflects different requirements and capabilities were catered to. Given that many group members were not digitally active, payment processes were difficult. Going forward, preparation of paper and postal based processes will be used.

Apart from two members, no tenants from Bulwell lunch club participated in the prioritisation discussions. This subgroup was challenging to engage due to digital inaccessibility, or they could not or were not willing to travel. Their involvement would have been insightful as some of the health issues they raised were exclusive to their group. Through ‘The Together Network’, greater effort will be made to reach out to these tenants by phone, post or in-person visits.

Despite efforts to encourage attendance and maintain engagement with tenants (i.e. newsletter and emails), there was considerable dropout throughout the process. Thirty-two tenants signed up to the group initially but only 15 participated in the prioritisation discussions. However, there was a core group of five or six, who took part in all stages of the process and remain engaged. This does, however, indicate that there were valuable perspectives that were lost. It is likely that the group that did participate was not reflective of the larger group, let alone the social housing community in Nottingham or elsewhere. Further, those who participated in the prioritisation discussions were mostly from NCCHS, female, heterosexual and white British between ages 60–79 (see Table 1). This may not be representative of Nottingham’s social housing population. Data from NCCHS, who provide homes to a fifth of Nottingham households, suggest that our data overrepresents females, tenants of 65 years of age or older and people who identify as disabled. It also suggests that our data underrepresents tenants younger than 24, but that we have well-represented those from black or minority ethnic backgrounds (see Table 1) [30]. It is possible that those who participated were more motivated or interested to do so as they had existing health issues, more free time (i.e., retired) or were more confident in voicing their opinions in groups settings. This could have excluded tenants without health issues, those who were employed/have other commitments or lacked confidence to join the group. It is also possible that the meetings being in English acted as a barrier to attendance for those who lacked proficiency in the language. However, translation services were offered, and no one expressed a need. Future PPI work should include a wider range of individuals from different backgrounds to ensure that their voices are heard – something which was difficult to achieve in the current work due to limited funding and there only being one researcher to carry out research activities. A more diverse group is something we hope to achieve with ‘The Together Network’.

Finally, the feedback survey provided some interesting discussion points. For example, one member felt like people attended meetings for monetary gain, rather than out of genuine interest. Another felt as though being part of this process had taught them about the “bigger picture”. Given that this feedback was collected after PPI meetings had come to an end, and the survey was also anonymous, there was no opportunity to explore these comments further. Future research could address them. Alternatively, if this process were to be repeated, for example in ‘The Together Network’, feedback could be collected via semi-structured interviews to allow probing and deeper exploration of answers (although, this would compromise anonymity, which can discourage honest responses).

In general, tenants were interested, enthusiastic and committed to being involved in public health research. The evaluation survey gathered positive feedback concerning the organisation and professional delivery of the process, enjoyment of group discussions and efficient communication from the researcher. The use of PPI meant that unique experiences were shared amongst strangers who related to each other over their identity as social housing tenants. Ideas were formulated which could not have been put forward by researchers without this lived experience. Given that the three final priorities were selected by tenants implies that this research meets the needs of people where we know there is need, thus closing a research gap, as to our best knowledge there is no literature that places tenants at the steering wheel of research and asks them what *they* want in terms of public health research. The current work has used processes whereby end-users of research are heavily involved in setting that very research agenda. Although there are areas for improvement, through discussion and prioritisation activities we have demonstrated that a collaborative, co-created research process with members of the public is a successful model to create meaningful outcomes and build the foundations for future work.

## Conclusion

This PPI work has demonstrated the value of involving the public in the pre-research stages. We have found that this selection of social housing tenants has concerns over three areas of their health and wellbeing more than others, that being: quality of housing, mental health issues and the healthcare service. Whilst the first two may be related, the latter is a problem most likely experienced by the public in general. Although there are concerns over the representativeness of the group, we have generally been met with commitment and enthusiasm. Future work should increase the diversity of the PPI group to ensure that voices of underrepresented populations are not lost. We recommend that other researchers continue to utilise PPI to ensure that research is conducted in areas it is needed the most.

### Electronic supplementary material

Below is the link to the electronic supplementary material.

Supplementary Material 1

Supplementary Material 2

## Data Availability

No datasets were generated or analysed during the current study.

## Abbreviations

HA: Housing Association
NCCHS: Nottingham City Council Housing Services
NCHA: Nottingham City Housing Association
NIHR: National Institute for Health and Care Research
PPI: Public and Patient Involvement
SAG: Social Advisory Group
