# Supplementary figures and images for: Bringing the voice of social housing tenants into shaping the health and care research agenda
Source: Res Involv Engagem. 2024 Aug 8;10:85. doi: 10.1186/s40900-024-00613-y (PMC11312242; doi:10.1186/s40900-024-00613-y)

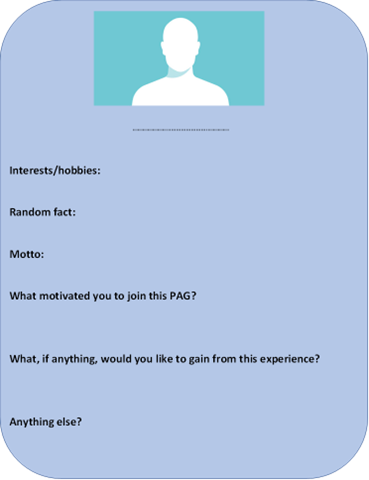

Supplement: Supplementary file 1 — Supplementary Material 1 [file 40900_2024_613_MOESM1_ESM.png]
