# Supplementary material for: Bringing the voice of social housing tenants into shaping the health and care research agenda
Source: Res Involv Engagem. 2024 Aug 8;10:85. doi: 10.1186/s40900-024-00613-y (PMC11312242; doi:10.1186/s40900-024-00613-y)
Supplement: Supplementary file 2 — Supplementary Material 2 [file 40900_2024_613_MOESM2_ESM.docx]

The list of health issues given to tenants to sort. ** indicates an NIHR funding theme

1. Few green spaces
2. Mental health e.g., depression, anxiety, apathy, stress
3. Poor relationship with police. There’s little to no intervention from them when problems arise. ‘Us vs them’ narrative
4. Few services or community groups for black people e.g., sickle cell support groups.
5. Smoking
6. Management issues - better and more efficient communication pathways between tenants and HAs/patch managers/council. More proactivity once HAs/patch managers contacted
7. Alcohol
8. Drugs
9. Treatment of disabled people
10. Fly tipping – disposing of rubbish/unwanted items on the street
11. Weight management
12. Poor safeguarding of children within social services
13. Stigma attached to social housing
14. Access to therapy/counselling
15. Quality of housing – mould, damp, poor insulation etc.
16. Access to urban areas
17. Few services within the home to support daily activities e.g., carers
18. Inaccessible environment e.g., pavements too narrow or poor quality, hilly surroundings, no lifts in places
19. First aid training (making it more inclusive of all skin colours)
20. Poor education about coping strategies to improve mental health
21. Loneliness/Isolation
22. Distrust of healthcare professionals e.g., doctors and dentists.
23. Lack of community activities and things to do e.g., lunch clubs, exercise groups, volunteering, socialising opportunities. No sense of community.
24. Poor healthcare services (doctor, dentist) – long wait times, poor care/environment in hospital, difficulties getting appointments, administrative issues, communication breakdowns
25. Sleep issues
26. Antisocial behaviour in the community
27. Male violence against women and girls**
28. The mental health and well-being of young women**
29. Eviction and homelessness**
30. The health of unpaid carers**
31. Highway infrastructure and its management on safety of pedestrians and cyclists and the uptake of active transport.**
32. Digital inclusion. Lack of access, skills and motivation for using digital technologies could contribute to worse health outcomes**
33. Health of migrants**
34. The use of performance enhancing drugs**
35. Illegal tobacco**
36. Air quality**
37. Onshore and gas exploitation on health/health inequalities**
38. Food insecurity**
39. Gang violence**
40. Gambling-related harm**
41. Vaccine uptake (where there is low uptake)**
42. School foods in SEN schools (Special Educational Needs)**
43. Suicide prevention**
44. Mental and physical health of the UK workforce**
45. Uptake of welfare benefits and vouchers for groups entitled to this support**
46. Domestic abuse**
47. Uptake of population screening programmes among under-served groups (e.g., newborn screening, non-cancer screening)**
48. Health of sex workers**
